# Irreversible oxidative post-translational modifications in heart disease

**DOI:** 10.1080/14789450.2019.1645602

**Published:** 2019-07-30

**Authors:** Tamara Tomin, Matthias Schittmayer, Sophie Honeder, Christoph Heininger, Ruth Birner-Gruenberger

**Affiliations:** a Institute of Pathology, Diagnostic and Research Center for Molecular Biomedicine, Medical University of Graz, Graz, Austria; b Omics Center Graz, BioTechMed-Graz, Graz, Austria; c Institute of Chemical Technologies and Analytics, Vienna University of Technology, Vienna, Austria

**Keywords:** Heart failure, oxidative stress, post-translational modifications, protein degradation, protein aggregation

## Abstract

**Introduction**: Development of specific biomarkers aiding early diagnosis of heart failure is an ongoing challenge. Biomarkers commonly used in clinical routine usually act as readouts of an already existing acute condition rather than disease initiation. Functional decline of cardiac muscle is greatly aggravated by increased oxidative stress and damage of proteins. Oxidative post-translational modifications occur already at early stages of tissue damage and are thus regarded as potential up-coming disease markers.

**Areas covered**: Clinical practice regarding commonly used biomarkers for heart disease is briefly summarized. The types of oxidative post-translational modification in cardiac pathologies are discussed with a special focus on available quantitative techniques and characteristics of individual modifications with regard to their stability and analytical accessibility. As irreversible oxidative modifications trigger protein degradation pathways or cause protein aggregation, both influencing biomarker abundance, a chapter is dedicated to their regulation in the heart.

## 1. Introduction

Heart failure (HF) is a complex, often lethal syndrome which is usually preceded by an acute onset of coronary artery disease (e.g. artery thrombosis) that obstructs the blood flow and thus reduces oxygen delivery to the heart. Oxygen deprivation can lead to ischemic/reperfusion injury of the myocardium, development of cardiomyopathy, myocardial infarction (MI) and other pathological conditions, many of which may result in a condition known as the failing heart, characterized by reduced ventricular filling or pumping capability [1].

Uninterrupted oxygen supply is of vital importance for normal heart function since the continuous energy demand of the heart is much higher compared to other tissues [2]. To meet these high energy requirements heart tissue contains a large number of mitochondria [3] contributing to oxidative stress. The myocardial oxidative metabolism is thus under strict control of enzymatic (e.g. superoxide dismutase, catalase) and non-enzymatic (e.g. tocopherol, ascorbic acid) anti-oxidative systems in order to neutralize potentially harmful side products of oxidative metabolism termed reactive oxygen and nitrogen species (RNOS) [4,5]. Small quantities of RNOS act as important signaling molecules that are involved in cellular stress sensing and response, regulation of mitochondrial function and immune response [6]. However, pathological conditions can dramatically alter the setting. Prolonged oxygen deprivation due to interrupted blood flow, so-called ischemia, can severely harm mitochondria and affect heart tissue contractility. The electron transport chain becomes dysfunctional resulting in decreased adenosine-triphosphate (ATP) production, followed by mitochondrial membrane depolarization and dysregulation of Ca^2+^ signaling [2,7–9]. If consequently blood flow is restored, the process of reperfusion causes further harm as ischemia-injured mitochondria and anti-oxidative defenses are unable to cope with the sudden boost in RNOS production. Increased RNOS induce further mitochondrial functional decline, trigger various signaling pathways, reduce contractile potential of cardiomyocytes and cause cardiac remodeling, promoting cellular injury and apoptosis of cardiomyocytes [1,3,10,11].

It has been attempted to establish specific and sensitive biomarkers predicting early stages of ischemic damage and cardiac remodeling in order to timely prevent HF. To date, several reliable protein/peptide-based prediction markers have been established and are already part of clinical routine [12]. The most prominent established protein markers for HF diagnostics are addressed in more detail within the next section of this review.

During the last decades, the advances in mass spectrometry enabled not only to monitor abundance of certain proteins or peptides as markers of HF but in addition to actually address local perturbations of the metabolome and proteome. These perturbations occur as a response to initial myocardial injury, followed by functional decline and concomitant rise of RNOS. In this regard oxidative stress induced post-translational modifications (oxPTMs) of proteins stand out as promising disease prediction tool [13,14]. As soon as exposed to oxidative stress, various amino acids of cardiac proteins are modified reversibly or irreversibly. The type and extent of modification can often signal the severity of RNOS exposure [13–15]. Some of these modifications are stable enough to be quantitatively monitored either directly or after derivatization. In addition, some of the irreversible oxPTMs can trigger specific protein degradation pathways and dysregulation of protein folding and clearance of misfolded proteins has been observed to correlate with different diseases [16]. Therefore, the second part of this review is dedicated to emerging protein markers with special focus on specific stable oxPTMs which could be used as readouts of cardiac oxidative stress and thus might aid early HF diagnosis and treatment. As proteins irreversibly modified by oxPTMs can only be removed by protein degradation pathways, one paragraph is dedicated to processes capable of breaking down these proteins, especially focusing on correlation of functionality of protein degradation pathways and heart disease.

## 2. Current protein-based biomarkers of heart failure

Given the high number and quality of extensive reviews on protein biomarkers in heart failure [12,17–19] this part of the review only covers two of the most well-established biomarkers in clinical practice: brain natriuretic peptide (B-type NP, BNP) and cardiac troponins T and I (cTnT and cTnI). Other common, but predominantly less specific, HF-related biomarkers are listed in Table 1. In the clinics, they are usually monitored by antibody-based methods. For further information, we refer to reviews on each biomarker specifically.

**Table 1.** Current clinical biomarkers of heart failure.

Biomarker	Pathophysiology	Detection method	Reference
BNP/NT-proBNP	Myocardial stretch	Immunoassay	Çavuşoğluet et al., 2019 [36]
Troponin T/I	Myocyte injury	Immunoassay	Wettersten & Maisel, 2015 [20]
Soluble ST2	Matrix remodeling	Immunoassay	McCarthy et al., 2018 [21]
Procalcitonin	Pulmonary infection	Immunoturbidimetry	Mockel et al., 2017 [22]
Copeptin	Neurohormonal activation	Immunoassay	Zhong et al., 2017 [23]
Galectin-3	Matrix remodeling	Immunoassay	Gehlken et al., 2018 [24]
Cystatin C	Renal dysfunction	Immunoturbidimetry	Breidthardt et al., 2017 [25]
C-reactive protein	Inflammation	Immunoassay	Swiatkiewicz & Taub, 2018 [26]

Brain natriuretic peptide (B-type NP, BNP) was originally identified in the brain, however later discovery of high BNP abundance in cardiac ventricles rendered it also a highly useful cardiac marker. By proteolytic cleavage of the 108-amino acid precursor proBNP_1-108_, the biologically active form BNP_1-32_ and the inactive N-terminal proBNP_1-76_ (NT-proBNP) are released [27]. Although NT-proBNP is not biologically active it is more stable than BNP, which has a half-life of only about 20 min [28,29]. Despite this fact, both BNP and NT-proBNP are today referred to as the ‘golden standard’ of biomarkers for HF [17].

After the so-called ‘Breathing Not Properly Study’ in 2002 [30] rapid measurement of BNP upon patient admission was suggested to verify HF diagnosis. It was claimed that BNP was ‘the single most accurate predictor’ of HF and elevated concentrations correlated with severity of HF. Furthermore, an independent study found a correlation between higher BNP levels on admission and in-hospital mortality, underlying the role of BNP as a biomarker in both diagnosis and prognosis of HF [31]. Thresholds for both BNP and NT-proBNP concentrations were established to ‘rule in’ and/or ‘rule out’ HF. For BNP a concentration of under 100 pg/ml represents a value for which HF is highly unlikely, while a concentration of more than 400 pg/ml suggests higher probability for HF. The upper thresholds for NT-proBNP are adjusted based on the patient’s age since NT-proBNP levels naturally rise with increasing age. NT-proBNP levels above 450, 900 and 1800 pg/ml mark the lower thresholds for HF diagnosis in the age groups of <50, 50–75 and >75 years, respectively [32]. Independent of age NT-proBNP level of <300 pg/ml is universally used to rule out HF. However, values of 100–400 pg/ml for BNP and 300–450, 300–900 or 300–1800 pg/ml NT-proBNP for the respective age groups of <50, 50–75 and >75 years represent concentration ranges where HF can neither be ruled out entirely nor diagnosed with certainty. Another caveat of using natriuretic peptide for diagnosis is the number of factors that can influence its levels. For example, obesity can lower BNP and NT-proBNP levels in patients [33], while renal dysfunction contributes to an increase in their concentrations [34]. In recent years the European Society of Cardiology has thus suggested that BNP peptides should only be used to rule out HF, but not to establish a diagnosis [35]. For a more extensive review on natriuretic peptides in clinical use, we refer to Cavusoglu et al. [36].

Next to BNP, measurement of cardiac troponin T or I levels represent another routine diagnostic tool for addressing myocardial infarction and HF. The troponin complex is an important part of both skeletal and cardiac muscle regulating muscle contraction [37]. Historically necrosis of cardiomyocytes was believed to be the prime reason for release of cardiac troponins, however it was recently shown that other mechanisms can lead to a detectable elevation of cardiac troponin levels [38]. Pioneer studies from the late 1990s revealed a strong correlation between increased levels of cTnT and cTnI and risk of HF, paving their way into clinical diagnostics [39,40]. Clinical incorporation of cTnT as a marker for myocardial infarction and HF was facilitated by the development of high-sensitivity assays for cTnT (hs-cTnT) revealing a link between troponin T and poor prognosis of HF patients [41]. It is important to mention that special care has to be taken when addressing cTnT levels of patients with skeletal myopathies. Likely due to cross-reactivity of skeletal and cardiac troponin isoforms in commercial immunoassays, it has been recently shown that skeletal myopathies can also result in an apparent elevation of cTnT concentration [42].

BNP and cardiac troponins provide easy and relatively quick markers for HF in the clinic. However, with different factors and comorbidities influencing these markers´concentration there is still a demand for more specific and more robust markers to emerge.

## 3. Oxidative post-translational modifications as potential biomarkers

**Figure 1.**
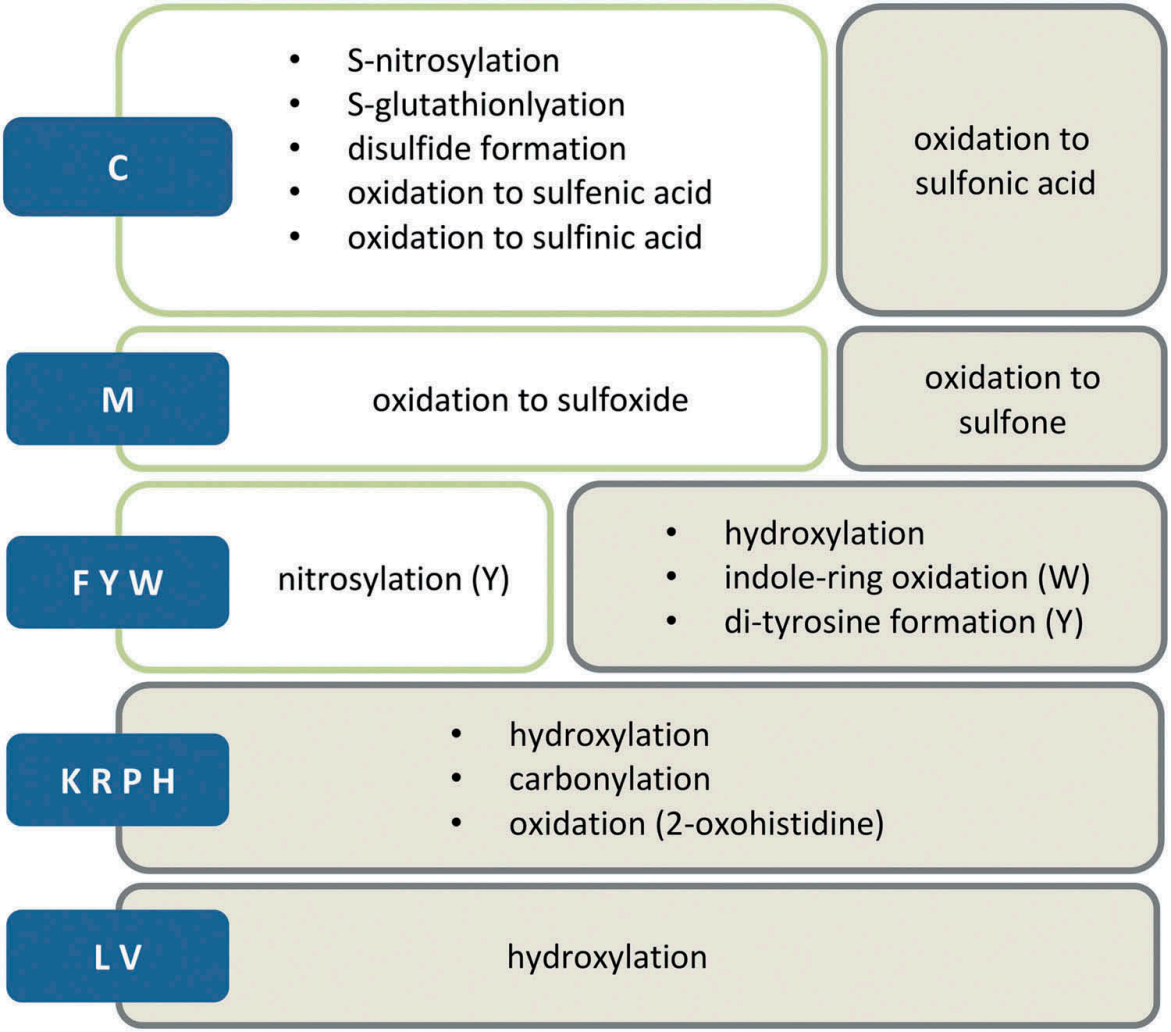
Most common oxPTMs. Blue boxes symbolize amino acids, green boxes represent reversible modifications and grey boxes irreversible ones.

One of the potential uprising HF biomarker resources are oxidative post-translational modifications (oxPTMs). As mentioned above, due to their highly active oxidative metabolism cardiomyocytes produce larger quantities of RNOS compared to other cell types, especially when exposed to ischemia and reperfusion injury. In addition to cardiomyocytes, infiltrated immune cells as well as epithelial cells of inflamed/injured vascular walls contribute to the increase of RNOS within heart tissue [13,43]. On cellular level, additional RNOS sources besides mitochondrial respiration include other oxygen-coupled enzymes such as oxidases (NAD(P)H and xanthine oxidase) (cyclo- and lipo-) oxygenases, uncoupled nitric oxide synthases (NOS) and cytochrome P450 enzymes [6,9]. Out of the variety of produced RNOS arguably the most toxic ones are hydrogen peroxide (H_2_O_2_), superoxide anion radical (O_2_^∙−^), hydroxyl radical (^∙^OH), nitric oxide (NO^∙^) and peroxynitrite (ONOO^−^) [6]. These RNOS, if not neutralized, can lead to a plethora of different reversible and irreversible oxPTMs on various amino acid residues. The most common reversible and irreversible oxPTMs are depicted in Figure 1 [6,13,44–46].

**Figure 2.**
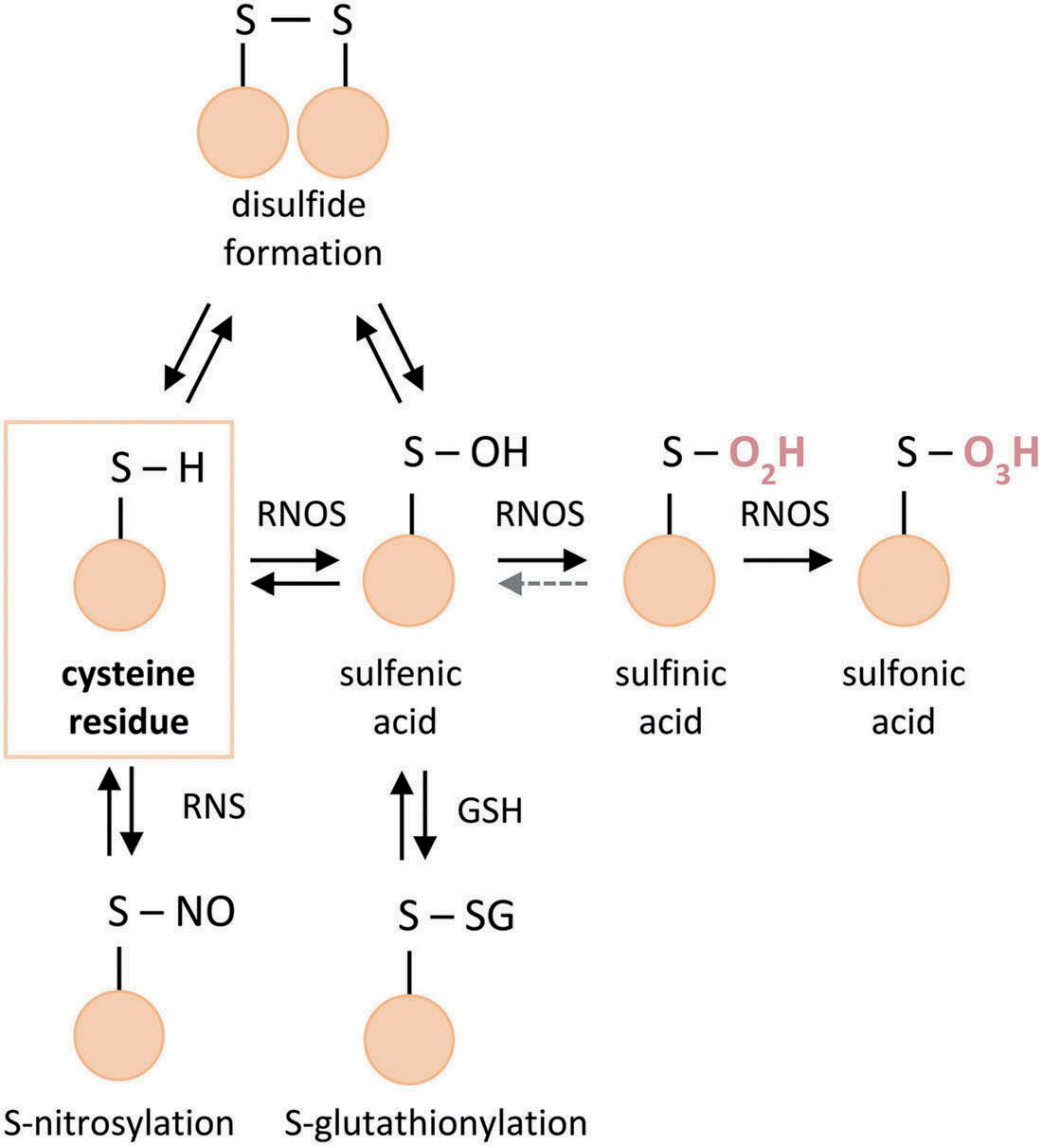
Overview of Cys modifications. Marked in red are irreversible oxPTMs. Grey dotted line: limited reversibility of sulfinic acid, RNOS – reactive oxygen and nitrogen species, RNS – reactive nitrogen species, GSH – glutathione.

Cysteine modifications have been reported to be the main oxidative signaling mediators, and the type of their modification to depend on the level of oxidative stress. Under less intense oxidative conditions the majority of Cys oxPTMs, such as S-nitrosylation, sulfenic acid and disulfide formation, are reversible and serve to modulate protein function [47]. However, prolonged exposure to high levels of oxidative stress leads to an increase in the oxidative state of Cys and renders the modification harder to reverse or irreversible (like in the case of sulfinic and sulfonic acid, respectively), which often causes loss of protein function [46,47] (Figure 2).

Addressing the reversible Cys reactions represents an ongoing challenge as free thiol residues are highly unstable and prone to artificial oxidation during sample preparation. In order to prevent post-sampling oxidation, most common approaches in redox proteomics involve initial blocking of free thiols with alkylating reagents such as iodoacetamide (IAA) or N-ethylmaleinimide (NEM) prior any further analytical steps [48]. Once all free thiols are blocked, higher oxidative states of Cys are either ‘trapped’ directly or selectively reduced back to free thiols and then subjected to a second alkylation step with isotopically and/or affinity-tag labeled alkylating reagents enabling enrichment and quantification of oxidized peptides. Another challenge when addressing reversible Cys oxPTMs is the stability of the given modification. For example, sulfenic acid, representing the initial step of Cys oxidation and an important modulator of protein function, is highly reactive and quickly interacting with surrounding amino acid residues, which makes sulfenylation a difficult modification to stabilize and detect. The speed of sample handling is of critical importance for sulfenic acid detection and the development of novel ‘trapping’ techniques, such as benzothiazine derivatives, is an ongoing effort [49,50]. For a better overview about specific methodologies for the analysis of reversible Cys modifications, we refer to Alcock et al. [50] and others [48,51].

The second sulfur containing amino acid, methionine, can also be easily oxidized by RNOS to methionine sulfoxide [52]. Although methionine sulfoxide is a chemically stable modification, methionine sulfoxide reductases are able to repair the oxidative damage [53]. Like in the case of cysteine, stronger oxidants can further push oxidation of methionine sulfoxide towards irreversible methionine sulfone formation [44].

Similar effects have been observed for tyrosine oxPTMs. While formation of 3-nitrotyrosine under mild oxidation/nitration conditions can affect protein function, exposure to severe oxidative stress levels leads to tyrosine dimerization triggering protein aggregation [45,47].

**Figure 3.**
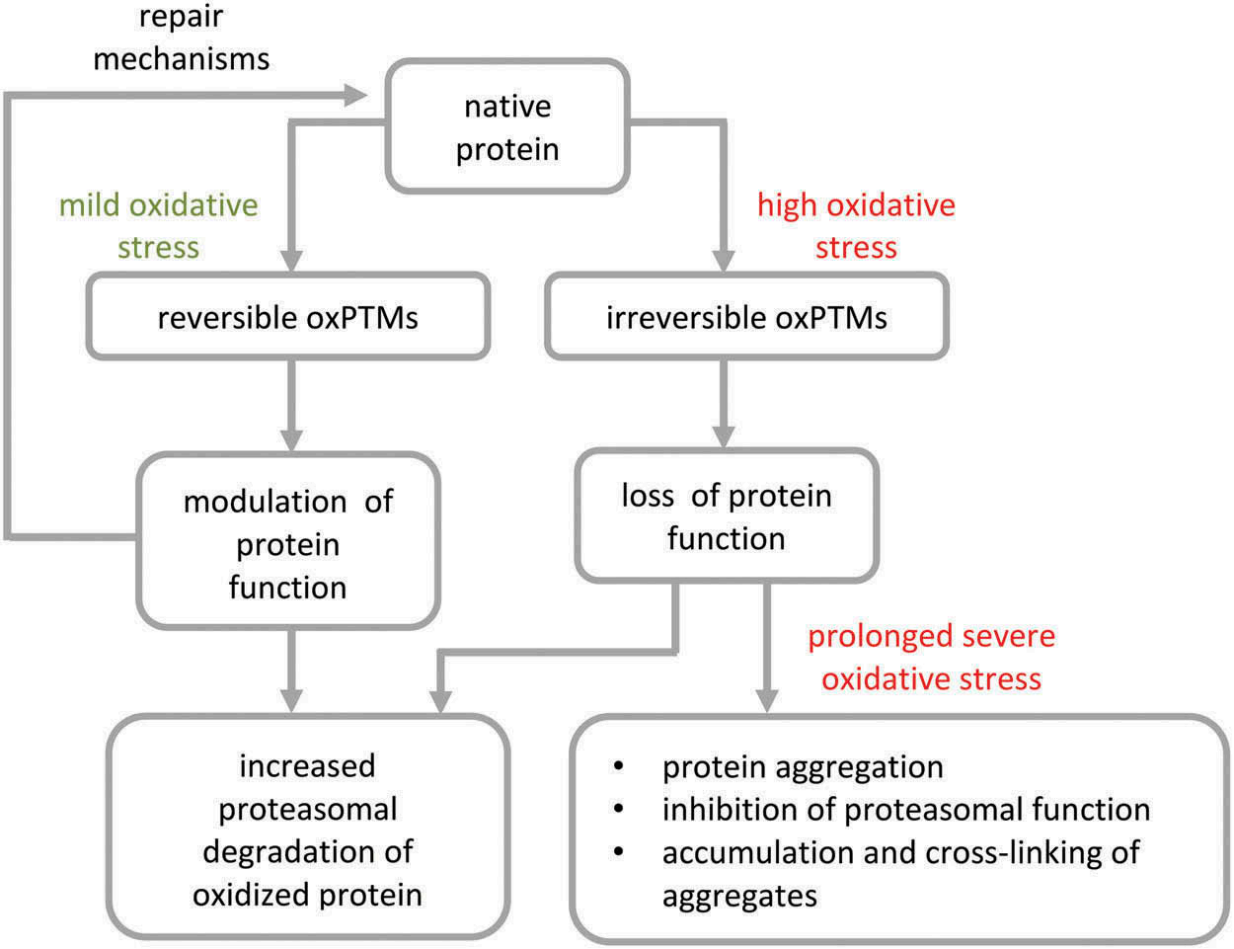
Effects of reversible and irreversible oxPTMs on protein function and stability. Reversible oxPTMs can either modulate protein function, get removed to restore native protein or induce degradation of damaged proteins, while irreversible oxPTMs usually cause loss of protein function and can either increase degradation or cause aggregation and accumulation of oxidized proteins due to inhibition of proteasomal activity.

Reversible oxPTMs, on the one hand, reflect the cellular oxidative and signaling state in a highly dynamic and sensitive manner. On the other hand, this property drastically limits their usefulness as stable biomarkers. Unlike reversible oxPTMs irreversible oxidative changes cannot be undone and can only be removed by protein degradation, which by itself can be seen as an individual oxidative stress response. One such example of an abundant irreversible oxPTM is carbonylation. Upon induction of oxidative stress in cardiomyocytes, generated RNOS react with lipids, especially those rich in polyunsaturated fatty acids. Oxidized lipid products are potent electrophiles which subsequently further react with various amino acids, predominantly nucleophiles such as lysine, arginine and histidine, causing carbonylation of proteins and triggering their proteolysis. While oxidation of lipids occurs minutes after induction of oxidative stress, carbonylation of proteins takes place in the timescale of approximately 1 h and is stable for several hours before the carbonylated protein is degraded [47,54]. An overview of effects of reversible and irreversible oxPTMs on protein function and stability is provided in Figure 3. Irreversible modifications and protein degradation pathways they trigger are described in more detail in the following sections.

### 3.1. Irreversible oxidative protein modifications

Some reversible oxPTMs do have important functional roles in redox signaling and can also protect oxidation sensitive sites of proteins from additional damage. However, when the level of RNOS stress exceeds the capacity of the cellular redox defense mechanisms, progressive oxidation of amino acid side chains may occur. The most obvious examples are the oxidation of cysteine beyond the level of sulfenylation (SOH), namely oxidation of the thiol to sulfinic and sulfonic acid (Figure 2).

**Figure 4.**
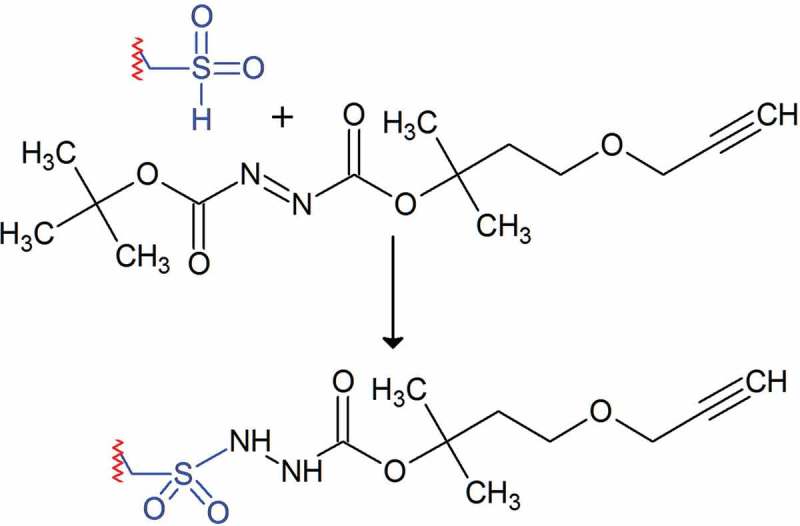
Chemoselective tagging of sulfinic acid residues employing electron-deficient diazenes [56].

For a long time, sulfinylation was believed to be an irreversible modification as sulfinic acid is a chemically stable modification. However, Biteau et al. identified sulfiredoxin (Srx1), an enzyme capable of reducing a specific sulfinic acid residue of the yeast peroxiredoxin Tsa1 in an ATP-dependent manner [55]. A similar system (hSrx) is active in humans and was also accepted to be highly substrate specific for a conserved site on peroxiredoxins I-IV [56]. However, a chemoproteomic approach recently carried out by the group of Kate Carroll identified more than 50 additional target proteins of hSrx [57]. They employed electron-deficient diazenes for chemoselective sulfinic acid nitroso ligation resulting in selective labeling of protein sulfinic acid residues (Figure 4). Differential isotopic labeling and biotin pulldown of labeled peptides in Srx-deficient versus -repleted mouse embryonic fibroblasts revealed approximately 20% of the 249 identified sulfinylation sites identified in both cell lines to be repaired by Srx activity. Among the discovered sulfinylated proteins were tyrosine-protein phosphatase non-receptor type 12, a phosphatase acting on constituents of the cytoskeleton and cell adhesion proteins, and Protein deglycase DJ-1, also known as Parkinson disease protein 7, which is a redox-sensitive chaperone which protects neurons from oxidative stress [58]. Interestingly, a site of the mitochondrial NADH-ubiquinone reductase NDUS1 was also reduced in Srx-deficient cells indicating the existence of another yet to be discovered sulfite reductase [57].

Along with the remaining ~80% of sulfinic acid sites not targeted by sulfiredoxins, all cysteines oxidized to sulfonic acid can be considered irreversibly oxidized because to the best of our knowledge, no repair mechanism for sulfonylation has been published. Both modifications are stable under common sample preparation and analysis conditions of bottom-up proteomics. Therefore they can, in theory, be analyzed employing routine LC-MS techniques. However, the low abundance of highly oxidized cysteines under basal conditions [59] can pose a challenge as, in contrast to most reversible oxPTMs, no simple chemical tagging and enrichment strategy is available. Only 1.5% of the amino acids assembling the human proteome are cysteine residues. Of those, an estimate of 1–2% is supposed to be oxidized under basal conditions. One possibility to overcome this hurdle is targeted MS methods trained on sites observed with data-dependent analyses under RNOS stress conditions [60]. Another approach is chromatographic enrichment based on the unique charge distribution of sulfinylated and sulfonylated peptides at acidic pH (≤2). Both, positive (anion exchange [61]) and negative selection (cation exchange [59]) of the negatively charged sulfonate group have been reported; the latter, in combination with a second hydrophilic interaction liquid chromatography step, reaches enrichment of up to 80% or an enrichment factor of greater than 2500 folds. The method was applied to map cysteine oxidation sites in a Langendorff-perfusion mouse model [62], an *ex vivo* model allowing examination of cardiac contractile strength and heart rate. As expected many irreversibly oxidized sites were only detected under ischemia/reperfusion injury conditions but not in non-ischemic time control (84 as compared to 24 sites, respectively). The majority of the ischemia/reperfusion sites were functionally annotated to the main clusters of myofilaments, mitochondrial proteins and oxidoreductases [59].

Similar functional protein clusters were also reported as targets of protein carbonylation, another type of irreversible oxidative modification in cardiovascular disease [63]. The targets reported in failing hearts with the highest number of carbonylated sites include alpha-actin and myocardial creatine kinase, both constituents of the cytoskeleton and energy metabolism, respectively.

**Figure 5.**
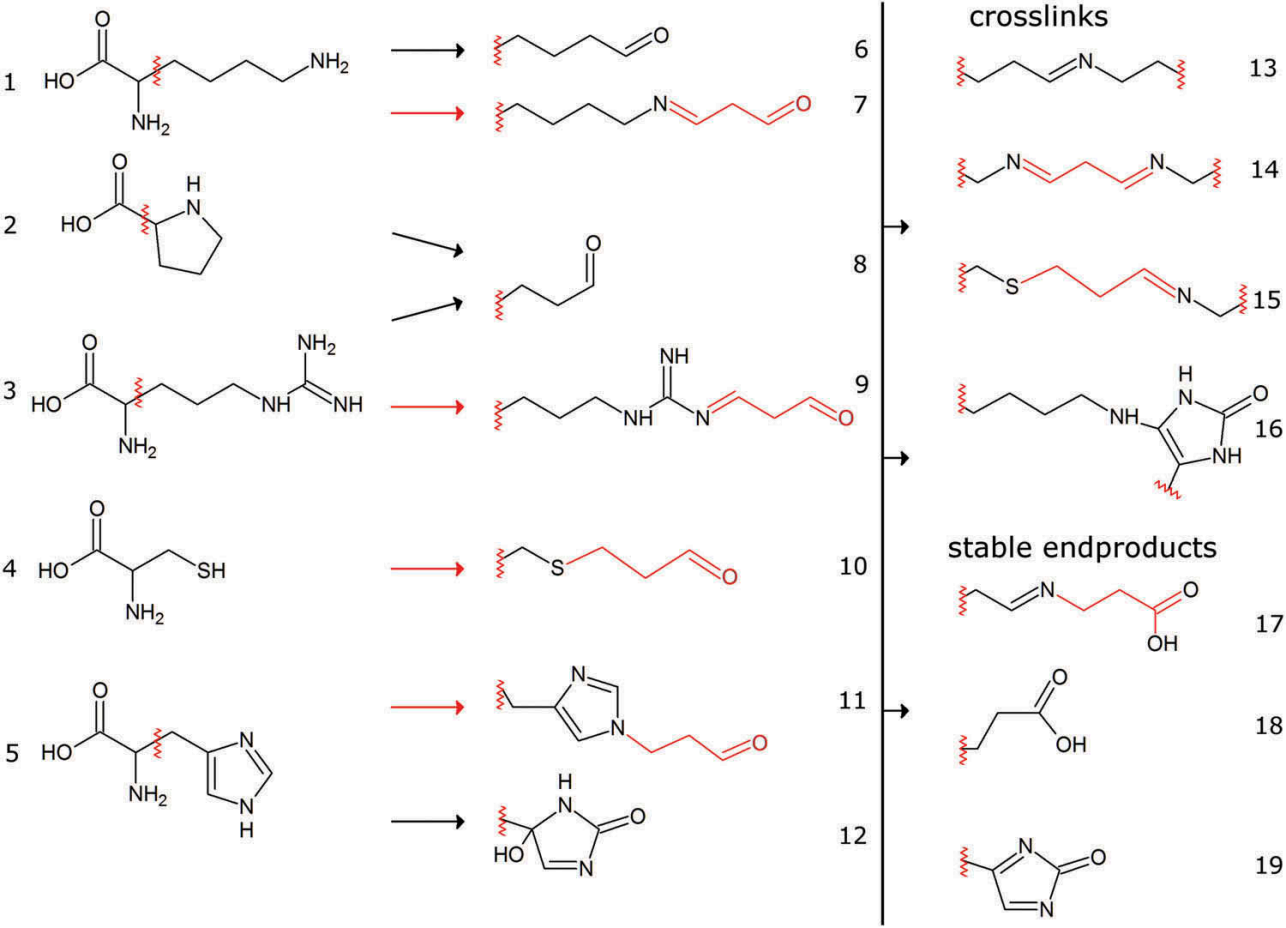
Selected amino acid carbonylation reactions. Amino acids (Lys (1), Pro (2), Arg (3), Cys (4), His (5)) can either react directly with RNOS (black arrows, 6, 8, 12) or with lipid peroxidation products (LPP, red arrows, 7, 9, 10, 11). Conjugates with malondialdehyde are depicted here as an exemplary LPP but many others including 4-hydroxynonenal and glycolysis side products such as glyoxal and methylglyoxal have been reported [53,71]. LPP can either react via Schiff base formation (7, 9) or by Michael-addition (10, 11). The reactive carbonyl groups can subsequently react with other amino groups of intra or intermolecular origin leading to a vast variety of different end products (13–16). Alternatively, hydrolysis, oxidation or enzymatic detoxification, e.g. via aldehyde dehydrogenases can yield stable end products with low reactivity (17–19).

Many studies of protein carbonylation rely on fluorescent or calorimetric labeling of carbonyl groups with hydrazines, e.g. dinitrophenylhydrazine (DNPH) [63,64]. However, without subsequent mass spectrometric characterization, these approaches cannot identify carbonylated proteins or the site of modification [65]. Carbonylation may either take place directly at the amino acid side chains of histidine, lysine, arginine, and proline, especially under transition-metal catalysis, or by reaction of amino acid residues with lipid peroxidation products (LPP). The two most abundant LPP causing protein carbonylation are malondialdehyde and 4-hydroxynonenal derived from oxidative degradation of lipid substrates. These LPP can react either directly with amino acid side chains to form a Schiff base or with nucleophiles in a Michael-addition mechanism (Figure 5) [13,54]. Irrespective of the mechanism, subsequent reaction of the product can lead to intra- and intermolecular crosslinks with other proteins (see next section). Alternatively, elimination of water [66], oxidation [67], further reaction with small molecules [68] or enzymatic detoxification [69] yield stable and less reactive products similar to advanced glycation end products which are derived from reactions of proteins with reducing sugars [70]. It has to be noted that only free carbonyls but not the crosslinked or the stable end products are detected by the widely employed DNPH approach. Therefore, only a thorough mass spectrometric analysis that takes the large variety of different possible products into account can potentially depict the entirety of protein carbonylation. Very recently, to our knowledge the so far most complete assessment of protein carbonylation by LPP was applied to a rat cardiomyocyte model by Griesser et al. [54], identifying constituents of the cytoskeleton, proteins of the extracellular matrix and proteins involved in cell adhesion as major targets.

Finally, irreversible oxidations also affect amino acids with aromatic side chains (Figure 1). Oxidation of aromatic amino acids can either take place in an enzyme-catalyzed manner, e.g. by monooxygenases and peroxidases, or via free hydroxyl radicals. While oxidation of phenylalanine yields tyrosine, further oxidation can either lead to the formation of protein-dihydroxyphenylalanine or to a di-tyrosine crosslink. Tryptophan can be oxidized to kynurenine via two intermediate steps by tryptophan dioxygenase or to 5-hydroxytryptophan via the serotonin biosynthesis pathway whereas random hydroxylations at various positions can occur in the presence of hydroxyl radicals [72,73]. A list of recent publications investigating irreversible oxidative modifications in the cardiovascular field is shown in Table 2.

**Table 2.** Recent proteomic studies of cardiac model systems reporting irreversible oxidative posttranslational modifications.

Irreversible oxPTM	Model or disease/tissue type	Affected pathways	Reference
Methionine sulfoxidation, sulfinic acid, sulfonic acid	Mouse hypertrophy/cardiac	Branched chain amino acid metabolism, fatty acid beta oxidation, TCA, glycolysis, creatine metabolism	Wang, J et al [109].
Oxidation of Tyr, Trp, Phe, Pro, Cys, Met, Asn, Asp, His, Lys,	Mouse/mitochondrial heteroplasmy	Oxidative phosphorylation/respiratory chain	Bagwan, N et al [124].
Carbonylation	Hamster cardiomyophathy/left ventricle	Respiratory chain, glycolysis, TCA	Ichihara, S et al [74].
Sulfinic acid, sulfonic acid	Mouse Langendorff perfusion/cardiac tissue	Myofilament, mitochondria, oxidoreductases, glycolysis, respiratroy chain, TCA	Paulech, J et al [59].
Sulfinic acid	General ROS stress/cultured cells, mouse tissues	Exosomes, oxidoreductases, cell adhesion, RNA processing, glycolysis, nuclear import, fatty acid beta oxidation	Akter, S et al [57].
Carbonylation (DNPH)	Mitochondria mouse heart and	TCA cycle, amino acid metabolism, respiratory chain	Carpentieri, A, et al [75].

### 3.2. Protein degradation triggered by oxidative damage

Proteolytic degradation of irreversibly oxidized proteins is crucial for the cell to maintain proper function [76,77]. As oxidative damage often leads to partial unfolding of proteins and concomitant exposure of hydrophobic patches otherwise buried within the protein, oxidized proteins tend to precipitate and/or aggregate [78]. If the cellular machinery does not readily degrade oxidatively damaged proteins, consequences are the accumulation of aggregates and cross-linking of the aggregates´ components eventually leading to the formation of large proteinaceous aggregates [77,79]. While protein aggregates are widely known for their pathophysiological role in neurodegenerative diseases their role in cardiovascular diseases and aging has become more apparent only in recent years [80–83].

In order to prevent the formation of protein aggregates the cell adapts to oxidative stress by increasing its proteolytic degradation capacity and substrate specificity towards oxidized proteins [84]. The primary machinery to degrade the bulk of improperly folded proteins as a first response is the proteasome, aided by the lysosomal system and some specialized proteases [77].

The ubiquitous and multi-catalytic proteasome consists of its 20S core of four heptameric rings arranged as two beta rings in the middle flanked by two alpha rings (α-β-β-α). The 20S core harbors the catalytically active β1, β2 and β5 subunits in the beta rings, which show caspase-l, trypsin- and chymotrypsin-like activities, respectively [85].

Several regulators attach to the 20S core and modulate its substrate specificity. For example, the 26S form of the proteasome consists of the 20S core with two 19S regulators attached and is responsible for maintaining protein turnover in homeostasis. Natively folded proteins that are no longer needed in the cell are ubiquitinated, recognized by the 19S subunits in an ATP-dependent manner and shuttled into the 20S subunits to be degraded. Interestingly the proteasome is highly potent in reacting to environmental cues [84]. Upon oxidative stress, the 26S proteasome readily dissociates into its 19S and 20S subunits [86], which is supported by the molecular chaperone HSP70. Oxidized proteins are then almost exclusively degraded ubiquitin/ATP-independently by the unbound 20S proteasome [85]. The corresponding decrease in 26S proteasome is also illustrated by elevated levels of ubiquitinated proteins [86] which are no longer degraded efficiently. The association of regulators, such as PA28αβ and PA28γ2, can have a positive influence on the degradation rate of oxidized proteins. These proteins facilitate opening of the alpha-ring gate enabling proteolytic degradation of oxidized proteins within the beta-core [87]. In addition, the exposed hydrophobic structures on oxidatively damaged proteins can also directly interact with hydrophobic binding domains on the 20S α-subunits to assist opening of the alpha-ring for entry of the damaged proteins into the catalytic core [88,89].

Furthermore, the proteasomal system is directly redox controlled through RNOS-induced modifications like carbonylation, S-glutathionylation, 4-hydroxynonenal modification, glycoxidation, phosphorylation and ADP-ribosylation [90]. For example, the proteasomal gating of the 20S core is directly influenced by S-glutathionylation of cysteine residues on proteasome α-subunits, and thus by the redox state of the cell. In the absence of enough reducing equivalents, thiol groups of cysteines can be activated forming oxidized functional groups (sulfenic acid), which react with glutathione (GSH) to generate S-glutathionylated proteins. Demasi et al. found that upon S-glutathionylation of α-5-C76 cysteine the 20S proteasome is present in open gate conformation and overall degradation of oxidized proteins is increased [78].

In case of extensive and prolonged oxidative stress, oxidatively damaged proteins are not degraded quickly enough and therefore accumulate, eventually forming covalently cross-linked bulky aggregates. Protein aggregates or damaged organelles that are too large for proteasomes can be degraded via the lysosomal degradation pathway, in which the damaged/oxidized contents are surrounded by a double membrane forming a vacuole termed the autophagosome. Autophagosomes fuse with the lysosomes. Break down of the damaged content occurs via acidic hydrolases inside these formed autolysosomes [90].

The highly oxidized aggregates are not only resistant to proteolytic degradation by the proteasome due to their bulky size but can also block the proteasome and thereby inhibit its function [83,91]. An example is the highly oxidized aggregate lipofuscin, which usually accumulates in lysosomes and is associated with aging and disease, being found in especially high abundance in neurodegenerative disorders [82,92]. Lipofuscin is able to block proteasome activity and further decrease the degradation of oxidized proteins [92]. Intriguingly, it has been only recently shown that lipofuscin is also accumulated in the aging human heart [82].

Protein aggregates and dysregulated clearance of damaged proteins are especially problematic in cardiomyocytes, as these cells are required to function in a coordinated manner to ensure the contraction ability of the heart. If aggregates become too large, contractile function can be compromised and lead to development of cardiomyopathy [81]. Aggregates have been shown to accumulate in heart tissue of both aged and hypertension-induced mice [83]. Quantitative proteomic analysis of these aggregates revealed the same set of proteins in both mouse models which were also connected to cardiovascular disease such as atrial natriuretic factor, apolipoprotein A-IV and tropomyosin [83]. Furthermore, soluble tropomyosin levels have been found to decline in aged rats, which might contribute to reduced contractility function [93]. A likely explanation for the observed reduction in tropomyosin in the soluble fraction is sequestration into aggregates. On the same line, proteasome subunits were found to be enriched in protein aggregates which correlates with the observed decrease in proteasomal activity with age [83]. Moreover, when hypertrophy was induced in mice it promoted the formation of aggregates in the heart and induction of autophagy [94]. Proteinaceous aggregates are also found in cardiomyocytes of patients with dilated and other cardiomyopathies [80]. One striking example of an aggregate-associated cardiomyopathy is the desmin-related form. The hallmark of desmin-related cardiomyopathy is the formation of aggregates consisting of desmin, an intermediate filament, and other proteins such as the small heat shock protein α-B-crystallin [95].

### 3.3. Specificity and predictive value of irreversible oxPTMs as potential markers of HF

Different types of oxPTMs, e.g. protein carbonylation, can arise in various environments and tissues. Hence they are not specific by themselves. However, the proteins on which oxPTMs occur can be unique for a certain tissue type, which renders oxPTMs on those proteins more disease specific. For heart failure prognostics, for example, carbonylation or irreversible cysteine oxidation of cardiac-specific proteins (such as myofilaments) can be used.

In this regard, it has already been shown that treatment of left ventricular cardiomyocytes with oxidants such as 2, 2′-dithio-dipyridine leads to an increase in oxidation of sulfhydryl groups of myofilament proteins (e.g. myosin light chain 1 and actin), which impairs contractile function and Ca^2+^ sensitivity [96]. This is further corroborated by the aforementioned report of global analysis of myocardial peptides after their enrichment for sulfinic and sulfonic modification. The authors identified 181 Cys-SO_2_H/SO_3_H sites from rat myocardial tissue subjected to physiologically concentrations of H_2_O_2_ (<100 μM) or to ischemia/reperfusion (I/R) injury. Out of the reported hits, seven Cys-SO_2_H/SO_3_H sites contained on six myofilament/cytoskeletal proteins were found in non-ischemic time-controlled samples, with the number increasing to 17 sites on 12 proteins following I/R. Accordingly some of the most modified proteins were as well different isoforms of actin and myosin [59]. The same was also true for carbonylation of tropomyosin and actin. Canton et al. reported around two-fold higher carbonylation of tropomyosin and actin in failing hearts compared to the controls. They also suggested that the degree of myofilament protein carbonylation (and S-nitrosylation) could have a predictive value for the extent of contractile impairment. The conclusion was drawn from the fact that higher levels of carbonylation were inversely correlated with left ventricular ejection fraction [97]. Furthermore, Brioschi et al. reported two cardiac-specific proteins that mainly underwent carbonylation in HF patients: M-type creatine kinase (M-CK) and α-cardiac actin [63]. In addition, it has been reported that diabetic hearts having higher degrees of cardiac protein carbonylation compared to non-diabetic hearts exhibited less residual left ventricular systolic function upon myocardial infarction [98].

Since irreversible oxPTMs of cardiac proteins are in majority local and not circulatory markers of heart failure it is noteworthy that also some irreversible oxPTMs of plasma proteins seem to be closely related to HF. Banfi et al. identified two proteins that mainly underwent carbonylation in the plasma of patients suffering from HF, namely α-1-antitrypsin and fibrinogen [99], suggesting a possibility that also oxPTMs of specific circular proteins might act as an independent marker of HF.

## 4. Summary and discussion

The major contribution of oxidative damage to cardiac myopathy was already recognized more than 20 years ago [100,101]. Several approaches can be employed to detect oxidative myocardial stress. First, general markers of inflammation such as c-reactive protein can be monitored as inflammation is important in the pathogenesis and progression of many forms of HF [102]. Next and by far more specific, secreted markers of myocyte stress such as b-type natriuretic peptide can be analyzed [103]. This has the advantage that already early disease stages will produce a detectable signal. However, as levels of BNP only reflect left ventricular (over)load they are highly variable between individuals and increase with age and inversely with the body mass index, which makes it difficult to interpret absolute BNP levels. Moreover, BNP levels can also increase as a consequence of head trauma [104,105] or acute ischemic stroke [106].

More specific markers for HF, on the other hand, require release of otherwise intracellular proteins into the circulation. This type of myocyte injury can occur after severe oxidative damage leading to cell death [107] and troponin I and troponin T are examples of biomarkers that may derive from this process. Unfortunately, these biomarkers are only applicable to aid diagnosis once acute coronary syndromes have already developed [102].

It is therefore of broad interest to find new biomarkers of cardiac myopathy with high specificity which can also be used to detect onset stages of the disease. A combination of independent established markers as suggested early on by Sabatine et al. [108] can improve the coverage of different types of heart failure but does not alleviate the problem of specific detection of early-stage disease. Promising new biomarkers based on oxidative post-translational modifications of cardiac proteins have been reported by several independent research groups [109–112]. A direct relationship of oxidative modifications of proteins and cardiac function was shown in murine animal models [113]. Interestingly, while some reversible modifications of especially cysteine residues were found to be cardio-protective [114], oxidative damage in the form of irreversible modifications was detrimental for cardiac function in all cases and either related to HF [63] or decreased cardiac capacity [115].

Several factors impact the incidence of oxPTMs of any given protein. First, as irreversible modifications accumulate over the lifetime of an individual protein, protein turnover is one decisive factor for the prevalence of oxidative damage. Especially macromolecules, such as the sarcomere or collagen, are prone to excessive oxidative damage which is one of the reasons their protein components are often among the most prominent hits identified in screens for oxPTMs. In healthy cardiac tissue, protein turnover occurs through the coordinated efforts of the ubiquitin/proteasome system, autophagy, and proteases such as calpain and caspases [116]. As discussed above, under RNOS stress partially unfolded proteins from the sarcomere can lead to activation of the ubiquitin-independent and less specific degradation by the 20S proteasome alone. Recruitment of the 20S proteasome to an insoluble protein complex, such as the sarcomere, can, in turn, lead to its inactivation and this process can halt the degradation of damaged proteins and promote further oxidative processes.

Another important factor determining the magnitude of oxPTMs is protein amount. The abundance of the sarcomeric proteins actin and myosin in cardiac tissue is very high [117] and the chance of RNOS reacting with these proteins is therefore increased as compared to less abundant proteins. On top of that, mass spectrometry is prone to oversample highly abundant analytes [118], which can overestimate the abundance of oxPTMs on these proteins.

Finally, local RNOS concentrations play a major role in the likeliness of oxPTMs occurring at a specific protein or site. Mitochondrial proteins operating in vicinity to the electron transport chain, a major contributor to cellular RNOS stress, are more likely to obtain oxPTMs. This is represented in the noticeable over-abundance of enzymes involved in the tricarboxyclic acid cycle such as malate dehydrogenase [113,119,120]. In enzymes harboring transition metals even intramolecular RNOS concentration differences can be observed as specific amino acids preferentially undergo oxidative modifications [72].

Aside from biological variations of RNOS species also methodological shortcomings can severely impact the observed oxPTMs. A crucial point not considered by most studies employing *in vitro* systems is the lower *in vivo* concentration of oxygen as compared to air. Physioxic conditions are tissue dependent but concentrations of 0.5–10% oxygen are commonly reported [121], which suggests a 2- to 40-fold overabundance of oxygen under commonly used cell culture conditions. The effect of long-term hyperoxic culturing on RNOS levels and expression of redox defense systems was recently investigated by Ferguson et al. A significant increase in RNOS levels and a plethora of adaptions to the hyperoxic conditions as compared to physioxia were found, including different levels of lipid peroxidation and increased antioxidant enzyme expression levels and activities [121].

Another potential methodological shortcoming is caused by ill-defined oxPTMs. While some oxidative mechanisms lead to a limited number of clearly defined end products, other modes of oxidation lead to diverse and extensive potential combinations. Not all these combinations can be detected by biochemical methods, e.g. DNPH can only react with the reactive intermediates of protein carbonylation but not with the stable end products. But also proteomic approaches suffer from the plethora of possible end products; allowing too many variable modifications leads to a combinatorial explosion of the search space and a concomitant loss of statistical power [122]. Novel search strategies have therefore been developed either based on dependent peptides [123] or wide precursor mass tolerance [124]. While these search strategies are better suited to deal with a large number of defined PTMs, they do not ameliorate the fundamental problem of undefined modifications such as random crosslinks between molecules. Upcoming methodologies employing combinatorial spectral libraries [125] or spectral clustering [126] offer a potential solution to this challenge.

Another analytical challenge derived from the low reactivity of the stable end products is selective enrichment of low abundant species. While efficient chemo-selective enrichment strategies have been devised for all but the least reactive oxPTMs [14,114,119,127], no such strategies exist for sulfonylated cysteines, cross-linked peptides or other terminal products of protein carbonylation. Two-dimensional chromatographic strategies can compensate for this lack to some extent but the majority of studies are still focused on less stable, more reactive oxPTMs for which well-established enrichment methodologies exist. However, precisely because of their increased reactivity these analytes are more susceptible to post-sampling error, especially considering the common variation of sample processing in a clinical setting [128].

Conclusively, the underlying mechanisms of redox signaling and reversible oxPTMs have been investigated thoroughly. However, as RNOS stress is omnipresent and hence also present in healthy tissue, reversible oxPTMs elevated above common levels can only provide evidence of an incident taking place within a limited time span of sampling. A more robust readout of continuous RNOS stress might be upregulation of redox defense systems or otherwise accumulated irreversible oxPTMs. While a significant amount of irreversible oxPTMs will be eliminated by specific protein degradation systems, the detectable fraction will correlate with the oxidative stress exceeding the capacity of the combined redox defense systems. Alternatively, being potentially more useful biomarkers for disease onset, upregulation of specific degradation pathways for proteins with irreversible oxPTMs can be monitored. Overall it is expected that proteomics will contribute to providing more accurate diagnostic and prognostic markers for heart disease in the near future.

## 5. Expert opinion

Current clinical biomarkers for HF suffer from lack of specificity and are not ideal for early detection of disease. A combination of independent markers may improve specificity and coverage of different types of HF. However, detection of early disease onset remains a challenge. Promising new biomarkers based on oxPTMs of cardiac proteins have been reported by several independent research groups. However, not all reversible oxPTMs are harmful; some can also be cardio-protective or related to signaling function. Oxidative damage in the form of irreversible post-translational modification of cardiac proteins, however, is universally detrimental for cardiac function and either related to HF or decreased cardiac capacity.

The occurrence of irreversible oxPTM of an individual protein is determined by its local environment, especially the local concentration of RNOS, its overall abundance, and the rate of its turnover. A caveat of *in vitro* studies based on cell culture models is that the concentration of oxygen *in vivo* is much lower than the one commonly used in cell culture resulting in increased reactive nitrogen or oxygen species levels and in adaptation to these hyperoxic conditions. This directly affects the type and abundance of oxPTMs of proteins. Thus, analysis of these modifications should be carried out *in vivo* or at least in physioxic conditions.

Major analytical challenges in detecting and quantifying oxPTMs are the large diversity of different possible molecular protein species produced by oxidation, often involving radical reactions, as well as their usually low abundance as compared to the unmodified protein species. Permitting too many variable modifications in standard proteomic data analysis workflows leads to a combinatorial explosion of the search space and a concomitant loss of statistical power. Novel search strategies based on dependent peptides or wide precursor mass tolerances, so-called open search, are better suited to deal with a large number of defined modifications. However, they are still unable to handle ill-defined modifications such as random crosslinks between molecules. Upcoming spectral clustering approaches of similar MS2 spectra have the potential to make these ill-defined modifications analytically accessible. While the low reactivity of irreversible oxPTMs potentially renders them very robust biomarkers it hinders their selective enrichment. Due to the lack of efficient chemo-selective enrichment strategies, multi-dimensional chromatographic strategies coupled to highly sensitive and fast mass spectrometry are required to account for their low abundance in different matrices with large dynamic range.

Owing to these combined technical difficulties most studies are still focused on less stable, more reactive and well-defined oxPTMs, for which well-established enrichment methodologies are in place and standard proteomic data analysis workflows can be employed. Because of their increased reactivity, these reversible oxPTMs are more susceptible to creating post-sampling artifacts. Moreover oxidative stress is also present in healthy tissue and increased levels of reversible post-translational modifications may not be specific for disease and only reflect one glimpse of a very dynamic process. Upregulation of the redox defense systems and/or accumulation of irreversible oxidative post-translational modifications are expected to be more robust readouts of continuous oxidative stress. The level of irreversible oxPTMs should correlate with oxidative stress exceeding the capacity of the combined redox defense and specific protein degradation systems. Alternatively, upregulation of specific degradation pathways for proteins with irreversible oxPTMs may be a promising early biomarker of HF. It is expected that recent developments in proteomics will contribute to providing more accurate diagnostic and prognostic markers for heart disease based on oxPTMs and protein degradation pathways in the near future.

## Article highlights

- Increased oxidative stress is linked to higher risk of heart disease and failure.
- As a result of oxidative stress, cardiac proteins are oxidatively reversibly or irreversibly modified by oxidative post-translational modifications (oxPTMs).
- Irreversible oxPTMs trigger protein degradation pathways.
- Monitoring irreversible, highly stable oxPTMs can aid heart disease prognostics.
